# How Does Influenza A (H1N1) Infection Proceed in Allogeneic Stem Cell Transplantation Recipients?

**DOI:** 10.5152/tjh.2011.74

**Published:** 2012-03-05

**Authors:** Sinem Civriz Bozdağ, Gülden Bozkurt, Pervin Topçuoğlu, Alpay Azap, Muhit Özcan, Nahide Konuk, Önder Arslan

**Affiliations:** 1 Ankara University, School of Medicine, Hematology Department, Ankara, Turkey; 2 Ankara University, School of Medicine, Infectious Diseases Department, Ankara, Turkey

**Keywords:** H1N1, Allogeneic transplantation

## Abstract

The clinical course of influenza A (H1N1) infection in allogeneic hematopoietic stem cell transplantation (AHSCT)recipients is not clearly known. We report 3 AHSCT recipients that were infected with influenza A (H1N1). Each of thepatients had a different hematological disease and was at a different post-transplantation stages. All the patients weretreated with oseltamivir, and zanamivir was switched to oseltamivir in 1 patient. All the patients survived without anycomplications. The course of swine flu can vary and progress with bacterial or other viral infections in immunosuppressedpatients.

## Abstract (tr)

H1N1 virüsünün allojeneik kök hücre nakli yapılan Hematoloji hastalarında kliniği ve seyri konusunda veriler oldukça yetersizdir. Biz bu olgu serisinde, Ankara Üniversitesi Tıp Fakültesi kök hücre nakli ünitesinde allojeneik nakil yapılmış ve Influenza A/ H1N1 infeksiyonu saptanmış 3 olgumuzu tartıştık. Üç hastamızın da farklı hematolojik malignite tanıları vardı ve nakilin farklı evrelerinde olan hastalardı. Tüm hastalara oseltamivir tedavisi verildi; bir hastada zanamivir tedavisine geçilmesi gerekti. Hastaların üçü de herhangi bir komplikasyon kalmadan tam iyileşme sağlanarak taburcu edildi. Influenza A/H1N1’e bağlı grip immunsupresif hasta gruplarında farklı seyirler gösterebilmekte, bakteriyel ve viral enfeksiyonlarla birlikte seyredebilmektedir.

## INTRODUCTION

Viral infections cause morbidity and mortality inpatients with hematological malignancies. The incidence and outcome of viral infections vary according tot he intensity and duration of T-cell depletion. Allogeneichematopoietic stem cell transplantation (AHSCT) recipients are highly susceptible to viral infections because ofimmunosuppression related to conditioning regimens,T-cell depletion, and graft versus host disease (GV HD) [1]. 

Influenza A (H1N1) in AHSCT recipients is associated with a wide range of symptoms. It can present with typical flu-like symptoms, such as fever, nausea, vomiting, diarrhea, and headache, and sometimes with atypical symptoms. Patients with malignancies can have more serious manifestations, including respiratory failure[2]. Prolongation of viral shedding has been observed in immunosuppressed patients; therefore, treatment of infection can be a major problem [3]. Oseltamivir and zanamivir have been approved for the treatment of influenza[4]. Herein we present 3 AHSCT recipients with different hematologic malignancies that were diagnosed with influenza A (H1N1) infection during different post transplant phases. Informed consent was obtained.

## CASE 1

A 23-year-old female acute myeloblastic leukemia (ALL ) patient underwent AHSCT from an HLA fully matched cousin during her second complete remission in March 2009. She relapsed 7 months post transplantation and was admitted to the hospital with pancytopenia. A second transplantation from an alternative donor was scheduled and the patient subsequently underwent the 2nd allogeneic transplantation from another HLA fully matched cousin following the FLAMSA regimen in December 2009. Thoracic high-resolution computed tomography (HRCT) results one month before the second transplantation were normal. Paranasal tomography results were compatible with sinusitis and she was treated with levofloxacin. Three days before transplantation she had an attack of febrile neutropenia and cefaperazone-sulbactam and amikasin were initiated. Her blood culture was positive for E. coli ESBL and cefaperazone-sulbactam was replaced with imipenem. The patient had herpes labialis and valacyclovir was changed from a prophylactic dose to a treatment dose. Antifungal prophylaxis with fluconazole was replaced with posaconazole.

The patent was non-febrile for 9 d, and then developed fever and cough 6 d post transplantation. PCR test results for H1N1 were positive and oseltamivir 75 mg BID was given. Posteroanterior lung X Ray showed right perihilar and paracardiac consolidation. Thoracic HRCT was repeated and the findings were consistent with fungal infection. Posaconazole was stopped and liposomal amphotericin therapy was started. The patient’s fever was controlled for 6 d, but recurred the same day that neutrophil engraftment was performed As the patient’s cough persisted at the end of 5 days oseltamivir treatment , we decided to continue 5 more days.. The patient’s attacks of fever were controlled and her cough was decreased by posttransplantation day 16.

## CASE 2

A 26-year-old male ALL patient underwent AHSCT from an unrelated donor during his second complete remission in July 2009. He did not have acute or chronic GV HD, but did have cytomegalovirus infection twice in 6 months, which was treated with ganciclovir. His immunosuppressive treatment was withdrawn 4 months post transplantation. As he had a history of disseminated fungal infection (Trichosporon) during chemotherapy, he continued to take voriconazole throughout the post-transplantation period. Antifungal therapy was stopped 2 months after the cessation of immunosuppressive therapy.

The patient had pancytopenia for 6 months and was admitted to the hospital in January 2010 with fever and cough. Piperacillin-tazobactam, oseltamivir, and voriconazole were started concomitantly. Posteroanterior lung X Ray showed reticulonodular infiltration in both lungs. PCR test results for H1N1 were positive. Oseltamivir 75mg BID was started with piperacillin-tazobactam and voriconazole. Thoracic HRCT showed ground glass density and a nodular appearance, which was considered indicative of viral and fungal pneumonia. Oseltamivir was withdrawn on d 6 of the treatment because of the patient’s fever persistence, and the development of anxiety, sinus tachycardia. Zanamivir was switched with oseltamivir for an additional 5 d, and then withdrawn. Sputum culture at the admission to the hospital, showed proliferation of Acinetobacter junii and the antibiotics were continued, according to the antibiogram. Piperacillin-tazobactam was replaced with imipenem and tigecycline.. The patient’s fever persisted and antifungal therapy was continued. Sputum cultures during the antibiotic and antifungal therapies were found positive for enterococcus and Stenotrophomonas maltophilia.The day after the results of bronchoalveolar lavage (BAL) was performed and the same pathogens were noted in the BAL culture. The patient’s fever was controlled using ciprofloxacin. The patient’s cytomegalovirus titer increased after resolution of fever. Gancyclovir was prescribed for two weeks until the negativity of CMV titer was achieved.

## CASE 3

A 35-year-old male patient underwent AHSCT from an HLA fully matched sibling in November 2008, following autologous transplantation due to refractory Hodgkin’s disease. He had stable disease for 8 months after AHSCT. He was diagnosed as zona zoster at the end of 8 th month and treated with acyclovir treatment. PET/CT control at the ninth month was consistent with progressive disease, chemotherapy was planned. Three days after the PET/CT control he was admitted to the hospital with fever, cough, and anorexia. PCR test results for H1N1 were positive. Posteroanterior lung X Ray was normal. Oseltamivir, 75 mg BID, piperacillin, and tazobactam were started 2 d after the onset of the fever and cough. The patient’s fever resolved during d 1 of the treatment and oseltamivir was withdrawn after 5 d of treatment.

## DISCUSSION

Patients with hematological diseases and AHSCT recipients are highly susceptible to influenza A (H1N1). Several studies investigated the clinical spectrum of influenza A (H1N1) infection in AHSCT recipients [5,6]; fever and cough are the most common symptoms. All the presented patients had fever and cough at the time of presentation; case 2 had more severe respiratory symptoms than cases 1 and 3. Viruses such as influenza can cause symptoms following contact with an infected person, but none of the presented patients reported such contact. The H1N1 virus most commonly affects individuals aged <25 years, such as the presented patients, but the HINI-associated mortality is higher among those aged 25-49 years. All the presented patients survived H1N1 infection. The use of antiviral agents is not recommended for healthy individuals, unless symptoms persist >48 h. Nonetheless, treatment should be initiated in transplantation recipients regardless of the duration of symptoms. Early administration of oseltamivir improves outcome [4]. Oseltamivir was initiated with 48 h of fever in cases 2 and 3, and after the 2nd attack of fever and cough in case 1. Antibacterial treatment was administered to the 3 presented patients and altered according to culture results. Cases 1 and 2 had influenza A infection concomitant with fungal and bacterial infections. Cases 1 and 2 were treated for fungal and viral infection, respectively. Each of the presented patients was at a different post-transplantation phase. The optimal dose of oseltamivir is a contentious issue that requires additional study. The presented patients were treated with oseltamivir 75 mg b.i.d., as they did not have gastrointestinal malabsorption due to chemotherapy or GV HD; however it is feasible to use higher doses in cases of high viral load and gastrointestinal absorption problems [4]. During the treatment of Case2 oseltamivir was switched to zanamivir due to the sinus tachycardia, anxiety side effects. There is no consensus concerning the optimal duration of oseltamivir treatment. As the symptoms in case 1 and 2 could not be controlled, treatment was administered for 10 d, but case 3 received oseltamivir for only 5 d.

The only way to prevented H1N1 infection is vaccination [7]. Post-transplantation immunosuppression remains a major problem for the immunogenicity of the H1N1 vaccine [8,9]. None of the presented patients had been vaccinated. The suspicion of our population about the effects and side effects of the vaccine was a problem to get over. In conclusion, based on our experience with the presented AHSCT patients with influenza A (H1N1) infection, we think that AHSCT patients may be highly susceptible to influenza, independent of the post-transplantation phase. All the presented patients received antiviral treatment and survived. The incidence and mortality has to be assessed in survey reports with large number of patients.

## CONFLICT OF INTEREST STATEMENT

The authors of this paper have no conflicts of interest, including specific financial interests, relationships, and/ or affiliations relevant to the subject matter or materials included.
